# First Molecular Detection and Characterization of Hemotropic *Mycoplasma* Species in Cattle and Goats from Uganda

**DOI:** 10.3390/ani10091624

**Published:** 2020-09-10

**Authors:** Benedicto Byamukama, Maria Agnes Tumwebaze, Dickson Stuart Tayebwa, Joseph Byaruhanga, Martin Kamilo Angwe, Jixu Li, Eloiza May Galon, Mingming Liu, Yongchang Li, Shengwei Ji, Paul Frank Adjou Moumouni, Aaron Ringo, Seung-Hun Lee, Patrick Vudriko, Xuenan Xuan

**Affiliations:** 1 National Research Center for Protozoan Diseases, Obihiro University of Agriculture and Veterinary Medicine, Obihiro, Hokkaido 080-8555, Japan; benards.benedicto4@gmail.com (B.B.); tumwebazeaggie@gmail.com (M.A.T.); JixuLi@hotmail.com (J.L.); eloizagalon@gmail.com (E.M.G.); lmm_2010@hotmail.com (M.L.); yongchangli8762017@outlook.com (Y.L.); Jishengwei0903@hotmail.com (S.J.); pfranck14@hotmail.com (P.F.A.M.); aringo2017@gmail.com (A.R.); 2 Research Center for Tropical Diseases and Vector Control, School of Veterinary Medicine and Animal Resources, Makerere University, Kampala 7062, Uganda; tayebwa.dickson@gmail.com (D.S.T.); josephjbvincent@gmail.com (J.B.); martinangwe@gmail.com (M.K.A.); 3 Department of Veterinary Pharmacy, Clinics & Comparative Medicine, School of Veterinary Medicine and Animal Resources, College of Veterinary Medicine, Animal Resources and Biosecurity, Makerere University, Kampala 7062, Uganda; 4 College of Veterinary Medicine, Chungbuk National University, Cheongju 28644, Korea; ggabheal@gmail.com

**Keywords:** hemoplasma, cattle, goats, PCR, Uganda

## Abstract

**Simple Summary:**

Hemoplasmas parasitize blood cells of several mammalian species including cattle, goats, and humans, causing infectious anemia in cattle and goats. Hemoplasmas are associated with significant production losses. However, no studies on bovine and caprine hemoplasmas in Uganda or the entire East African region have been carried out. In this study, we utilized molecular techniques to investigate the occurrence of hemoplasma species in goats and cattle from Uganda. Four hemoplasma species were identified in cattle and goats, with goats showing a higher risk for hemoplasma infection than cattle. This is the first molecular evidence of hemoplasmas in cattle and goats from Uganda and the entire east African region.

**Abstract:**

Hemoplasmas (hemotropic mycoplasmas) are small pleomorphic bacteria that parasitize the surface of red blood cells of several mammalian species including cattle, goats, and humans, causing infectious anemia. However, studies on hemoplasmas have been neglected and to date, there are no studies on bovine and caprine hemoplasmas in Uganda or the entire East African region. In this study, a polymerase chain reaction (PCR) assay targeting the 16S rRNA gene was used to investigate the presence of hemoplasma in 409 samples (cattle = 208; goats = 201) collected from Kasese district, western Uganda. Results showed that 32.2% (67/208) of cattle samples and 43.8% (88/201) of goat samples were positive for hemoplasmas. Sequencing analysis identified *Candidatus* Mycoplasma haemobos and *Mycoplasma wenyonii* in cattle, while *Candidatus* Mycoplasma erythrocervae and *Mycoplasma ovis* were identified in goats. Statistical analysis showed that goats were at a higher risk of infection with hemoplasmas compared with cattle. To the best of our knowledge, this is the first molecular evidence of hemoplasmas in bovine and caprine animals in Uganda and the entire east African region.

## 1. Introduction

Hemoplasmas, also known as hemotropic mycoplasmas, are small pleomorphic, wall-less, non-cultivatable bacteria that parasitize the surface of red blood cells of some mammalian hosts including cattle, goats, and humans [1,2]. Originally, hemoplasmas were classified as members of two genera (*Eperythrozoon* and *Haemobartonella*) of the order Rickettsiales, but years ago, evidence from phylogenetic studies of their 16S rRNA gene sequences led to the reclassification of these organisms as members of the genus *Mycoplasma* [3,4]. In animals, disease manifestations due to hemotropic mycoplasma infection are commonly reported in association with drug- or virus-induced immunosuppression, with stressors such as poor nutrition, pregnancy, or lactation, or with concurrent infection with other more virulent pathogens [5,6,7].

In cattle, there are two main hemoplasma species that have been identified: *Mycoplasma wenyonii* (formerly *Eperythrozoon wenyonii*) and a provisional species *Candidatus* Mycoplasma haemobos [8,9,10]. Infected cattle can develop clinical signs such as anemia, transient fever, lymphadenopathy, anorexia, weight loss, and decreased milk production, although in most of the animals, the infection remains subclinical [11]. The possible transmission routes of bovine hemotropic mycoplasmas include by vectors such as fleas, hard ticks, and mosquitoes, or by direct contact with contaminated blood, but the epidemiology is still poorly understood [12,13].

In goats and other small ruminants, *Mycoplasma ovis* and *Candidatus* Mycoplasma haemovis are the most common agents of hemoplasmosis. These hemoplasma species frequently cause hemolytic anemia and reduced exercise tolerance in sheep with acute infection [1,14,15,16]. Infected goats have less pronounced clinical signs characterized by low bacteremia, thus, eventually becoming persistent carriers [17,18]. Natural transmission occurs by blood sucking arthropods such as ticks and mosquitoes, while mechanical transmission is by fomites [19]. The negative impact of hemoplasma infections in small ruminant production is considerable because of mortality and productivity losses in chronically infected animals [14,20].

The livestock sector contributes 5% to Uganda’s national gross domestic product (GDP) and sustains food security and livelihood for 75% of the households [21]. Uganda cattle and goat populations are estimated at 14.5 and 15 million, respectively [22]. To the best of our knowledge, molecular detection of bovine and caprine hemoplasmas has never been performed in Uganda or the entire East African region, despite the potential economic impact of hemoplasmas on livestock production. Our search for documented studies on hemotropic mycoplasmas in ruminants in Africa yielded only two reports: one on Nigeria cattle [23] and another on wild buffalo (*Syncerus caffer*) in Mozambique [24]. Therefore, the aim of this study was to utilize polymerase chain reaction (PCR) assays and sequencing analysis to investigate and confirm the presence of bovine and caprine hemoplasma species in Uganda.

## 2. Materials and Methods

### 2.1. Ethical Statement

The study was approved by the Ethics Committee of Ministry of Agriculture Animal Industry and Fisheries (MAAIF) Uganda (IHVC-PRODUCTS No. 00044840). Consent was obtained from farmers before sampling their animals. All experimental procedures were carried out according to the ethical guidelines on the use of animal samples of the Obihiro University of Agriculture and Veterinary Medicine (experimental approval ID: 19-15; DNA experimental ID: 1724-3).

### 2.2. Study Area

**Figure 1:**
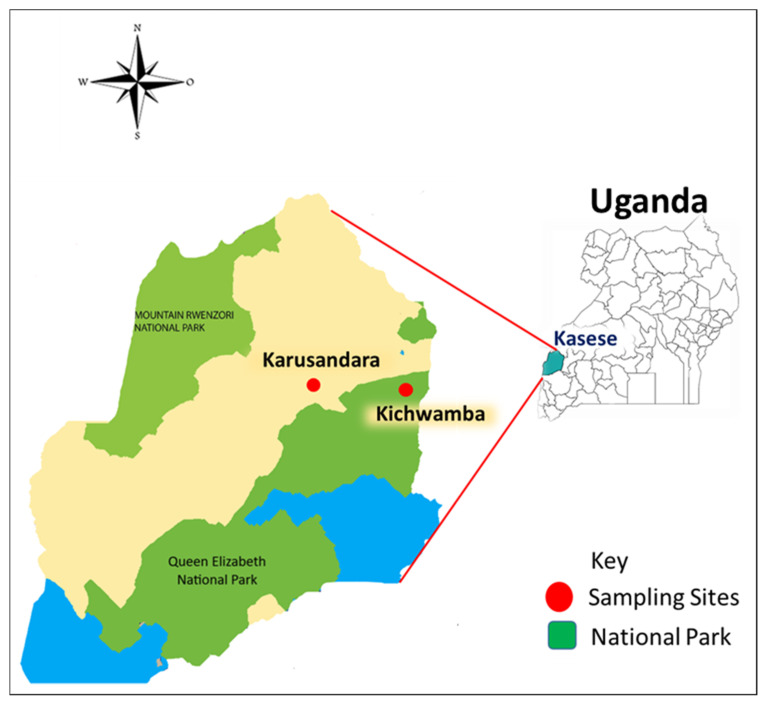
Map of Kasese district (located in western Uganda) showing the location of the two sampling subcounties (Karusandara and Kichwamba). The figure was generated and modified using GIMP 2.8.10 (https://www.gimp.org). Maps were obtained from d-maps.com (https://d-maps.com/index.php?lang=en).

We conducted a cross-sectional study between May and June 2019 and collected blood from cattle and goats in farms located in Kasese district—i.e., Karusandara subcounty, located 6 km away from Queen Elizabeth National Park (QENP), and Kichwamba subcounty, located within QENP boundaries (Figure 1).

Kasese district is made up of 21 subcounties, 130 parishes, and 730 villages. According to the Köppen Climate Classification, the district has a tropical savanna climate. The average temperature for the year in Kasese is 23.8 °C. March and December are the warmest and coolest months with average temperatures of 24.5 °C and 23.1 °C, respectively. Kasese’s annual average amount of precipitation is 883.9 mm. April and January are the months with the most and least average precipitation of 127 mm and 27.9 mm, respectively. In terms of liquid precipitation, there are an average of 146 days of rain, with the most rain occurring in October, with 19 days of rain, and the least rain occurring in January, with 7 days of rain. This area was chosen due to many pastoralist farmers who co-graze their livestock with wildlife at the QENP interface, making it a high risk for cross-species pathogen transmission.

### 2.3. Study Design

The two subcounties above were randomly selected as sampling sites. For cattle samples, 9 to 17 heads of cattle per farm were randomly selected, depending on the herd size. The selected animals were restrained in a crush and examined for parameters like age, sex, and body condition score. In total, 16 farms were included yielding a total of 208 cattle. The blood samples were collected through puncture of the caudal vein and letting blood into tubes containing ethylenediaminetetraacetic acid (EDTA) anti-coagulant. For goats, a total of 19 farms were selected, from which 201 goats were sampled by puncture of the jugular vein and letting blood into EDTA tubes. The collected samples were kept in a cool box and transported to the Research Centre for Tropical Diseases and Vector Control (RTC), School of Veterinary Medicine and Animal Resources, Makerere University, Uganda, for DNA extraction.

### 2.4. DNA Extraction and PCR Amplification Procedures for Detection of Hemoplasmas

The DNA was extracted from 200 µL of whole blood using QIAamp DNA Blood Mini Kit (Qiagen, Hilden, Germany) in accordance with the manufacturer’s instruction. The extracted DNA was then transported to the National Research Centre for Protozoan Diseases (NRCPD), Obihiro University of Agriculture and Veterinary Medicine, Japan, and stored at −30 °C until use. The DNA samples were screened with the F2R2 set of primers, which targets the 16S rRNA gene of various hemotropic mycoplasmas including *M. haemofelis*, *M. haemocanis*, *Candidatus* M. haemominutum, *Candidatus* M. haemoparvum, *M. wenyonii* and *Candidatus* M. haemobos, *M. ovis*, and *Candidatus* M. erythrocervae, among others [9,25,26] (Table 1). The thermocycling protocol for F2R2 primers used in this study was that described by Jensen et al. [26] with slight modifications, whereby the initial denaturation was set at 95 °C for 2 min, followed by 40 cycles of denaturation at 95 °C for 1 min, then annealing at 60 °C for 1 min, extension at 72 °C for 30 s, and final extension at 72 °C for 7 min.

The reaction mixture had a final volume of 10 µL containing 1 µL of 10× ThermoPol^®^ *Taq* reaction buffer, 0.2 µL of dNTP mix, 0.2 µM of each primer, 0.05 µL of *Taq* DNA polymerase (all from New England BioLabs, Ipswich, MA, USA), 1.5 µL of DNA template, and 6.85 µL of double-distilled water. The positive samples used in the study were previously confirmed hemoplasma-positive (*M. ovis*, *M. wenyonii*, and *Candidatus* M. haemobos) DNA samples [27], while double-distilled water was used as the negative control. The thermocycling conditions used were those of previous studies, with the specific annealing temperatures shown in Table 1.

### 2.5. Cloning, Sequencing, and Phylogenetic Analysis

A total of 25 positive samples (cattle = 15; goats = 10) were randomly selected for sequencing. Another set of primer, the HBT primers, was used due to the longer target (~595 bp) compared to the F2R2 primer set, which amplifies <200 bp nucleotide sequence (Table 1). Briefly, PCR products run on agarose gel were purified using QIAquick Gel Extraction Kit (Qiagen), and DNA was quantified using NanoDrop spectrophotometer (Thermo Fisher Scientific, Ipswich, MA, USA). The purified PCR products were cloned into a vector following the commercial protocol of pGEM^®^-T Easy Vector (Promega Corporation, Madison, WI, USA) and then transformed into *Escherichia coli* DH5*α* competent cells. For each sample, four clones with the expected insert (as confirmed by colony PCR) were cultured and purified using Nucleospin^®^ Plasmid QuickPure Kit (Macherey Nagel, Düren, Germany). Sequencing analysis was done with BigDye^™^ Terminator v3.1 Cycle Sequencing Kit (Applied Biosystems, Waltham, MA, USA) using ABI Prism 3100 Genetic Analyzer (Applied Biosystems). The resulting nucleotide sequences were analyzed by GenBank BLASTn search, and percent identities were calculated using EMBOSS Matcher, an online program (https://www.ebi.ac.uk/Tools/psa/emboss_matcher/). The nucleotide sequences generated were deposited in NCBI GenBank database.

### 2.6. Statistical Analysis

Comparisons of the infection rates between cattle and goats and risk factors were performed using Pearson’s chi-square test and Fisher’s exact test. A *p*-value of <0.05 was considered statistically significant.

## 3. Results

### 3.1. Detection Rates in Cattle and Goats

**Figure 2:**
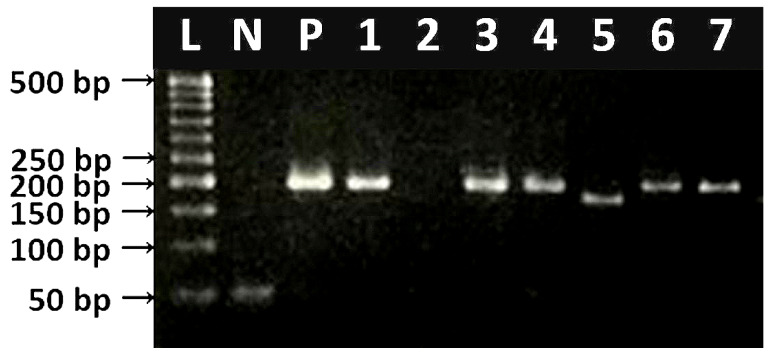
Agarose gel electrophoresis (2% agarose), showing amplified DNA (F2R2 primers, 175–200 bp) for 16S rRNA gene of hemoplasmas. L = 50 bp DNA ladder; N = negative control; P = positive control (*Mycoplasma ovis*); 2 = negative sample of uninfected animal; 1, 3–7 = genomic amplicons of infected animals.

A total of 409 blood samples were collected from cattle (n = 208) and goats (n = 201) from two subcounties in Kasese district in Uganda (Figure 1). Additional information concerning sex, age, breed, body condition score, and herd size was obtained (Supplementary Table S1). Overall, 37.9% (155/409) tested PCR-positive for hemoplasmas. Of the 155 positive animals, 67 (32.2%) and 88 (43.8%) were cattle and goats, respectively. The different band sizes obtained were as seen in Figure 2.

### 3.2. Risk Factors

Hemoplasma-infected cattle and goats did not exhibit any clinical signs attributable to hemoplasmosis, such as pale mucous membranes, transient fever, lymphadenopathy, and anorexia. However, goats were found to be at a higher risk (*p* < 0.05) of infection than cattle, as seen in Table 2.

Similarly, male cattle had a significantly (*p* < 0.05) higher hemoplasma detection rate than female cattle (Table 3). The prevalence of hemoplasma species in goat samples collected from Karusandara subcounty was significantly higher than that collected from Kichwamba subcounty (Table 3). However, there was no significant difference in the hemoplasma detection rate between the cattle samples collected from the two subcounties. In addition, other factors like breed, age, body condition score, and herd size were not significantly associated with infection in both cattle and goats (data not shown).

### 3.3. Identities of Obtained Sequences

Identification of hemoplasma species was further demonstrated by sequencing analysis of the partial sequence of 16S rRNA gene of 25 hemoplasma-positive samples (15 cattle samples; 10 goat samples). All the sequences obtained in this study were deposited in GenBank and assigned temporary accession numbers, as seen in Table 4.

For the cattle samples, 7 sequences (CMh1-9) were 97 to 100% identical to each other, wherein 5 out of these 7 sequences (CMh 1–4,7) exhibited >97.3% identity with *Candidatus* Mycoplasma haemobos from Tokachi, Japan (EU367965). The other 6 cattle sample sequences (Mw1–6) were 100% identical to each other and also exhibited 100% identity with *M. wenyonii* strain isolated from a water buffalo in the Philippines (MT241312) and from cattle in Cuba (MG948627). For the goat samples, 8 sequences (Mo1–8) were 100% identical to each other and exhibited 100% identity with *M. ovis* isolated from goat in China (KU983745), sheep in Turkey (MF377462), and a human isolate in the USA (KF313922). The other remaining goat sample sequence (CMe1) also exhibited 100% identity with *Candidatus* Mycoplasma erythrocervae isolates from sika deer in Japan (AB558897 and KF306251).

## 4. Discussion

To our knowledge, this study provides the first molecular evidence of hemotropic mycoplasmas in cattle and goats in Uganda and throughout the east African region. A significantly higher rate of hemoplasma was detected by PCR in the sampled goats than in the cattle. This is probably due to epidemiological factors such as host, environment, and the difference in the hemoplasma species infecting cattle and goats [1].

The detection rate of bovine hemoplasma species in this study (32.2%) is higher than that reported in Japan, where detection rates of 21.8 and 16.7% were found [9], but is lower than those reported in studies from Nigeria [23], Cuba [28], Brazil [29], and eastern Hokkaido, Japan [30], where overall detection rates of 67.0%, 53.0%, 64.2%, and 64.7% were reported, respectively. In goats, the detection rate obtained in this study (43.8%) was comparable to those in China [13] and Brazil [18], where detection rates of 41% and 39.3% were reported, respectively. However, the detection rate in the current study was much higher than those reported in Turkey at 6.2% [16] and USA at 18% [31]. Factors such as geographic location, sample size, animal age, environmental conditions, presence of vectors, and animal production systems can be possible causes of the observed disparity in the detection rates between the present work and previous studies [16].

Studies on bovine hemoplasma infection in Japan found significant association between reduced productivity and hemoplasma infection [32]. In another study, examination of blood parameters revealed significantly lower red blood cells, hemoglobin, and packed cell volume levels, and a higher mean corpuscular volume in infected cattle than in non-infected cattle [20]. Therefore, the detection of these hemoplasma species in Ugandan cattle raises concern about possible economic losses in livestock production that farmers could incur, and economic impact assessment should be included in future studies.

In this study, a higher hemoplasma detection rate was recorded in male cattle than in female cattle. Sex has previously been associated with hemoplasma-positivity in small ruminants [18,23,27], but this phenomenon has not been observed in cattle. Some literature suggests that female animals normally go through pregnancy and lactation stress, which tend to stress and lower their immunity, making them more susceptible to infections [33]. In a previous study on the effect of chronic hemoplasma infection on cattle productivity [32], no significant relation between hemoplasma infection and sex was observed. However, the small sample size of male cattle in this study may not strongly support our finding; thus, further studies using equal sample sizes should be conducted to understand whether hemoplasma infection is associated with sex in cattle.

When the results from the two subcounties were compared, the goat samples from Karusandara subcounty (an area 6 km away from the park) had a significantly higher hemoplasma detection rate than those from Kichwamba subcounty (an area within the national park). On the other hand, no significant association was seen with the cattle samples collected from the two locations. In addition to the wildlife-livestock interaction reported by the farmers, the goats in Karusandara subcounty were grazed communally under a free-range system, which could be a risk factor for infection with hemoplasmas. *M*. *ovis,* which is the main goat species also identified in this study, can infect goats, sheep, and deer species [34]. In addition, current literature indicates that hemoplasmas can infect a variety of wild animals including buffalo, rodents, deer, cheetah, jaguar, bear, opossum, raccoon, bats, and non-human primates [35]. Wildlife–livestock interactions have previously been reported in this area surrounding Queen Elizabeth National Park with empirical evidence [36]. However, there is still limited data regarding the epidemiology of hemoplasmas. Therefore, further studies are recommended to understand the role of the wildlife-livestock interface in the transmission of hemotropic mycoplasmas.

Nucleotide sequences obtained from cattle were 100% identical to *Candidatus* Mycoplasma haemobos and *M. wenyonii.* This was not by surprise, since these two species are the only distinct hemoplasma species that have been identified affecting cattle to date [8]. In goats, we identified *M. ovis*, the most common species in small ruminants [1,14,16,30], and *Candidatus* Mycoplasma erythrocervae, which infects deer [37]. Since the samples in this study were collected from a wildlife-livestock interface area (Figure 1), the detection of *Candidatus* Mycoplasma erythrocervae in goats suggests a possible cross-species transmission, since there is evidence of wildlife-livestock interaction in this area [36]. Furthermore, hemoplasmas seem to exhibit a non-specific host preference, for instance, the *M. ovis* genotype in this study showed 100% identity to the *M. ovis* previously isolated from a symptomatic human (KF313922) in the USA [38]. In another study on hemotropic mycoplasma species in patients with or without extensive arthropod or animal contact [2], the prevalence of hemotropic mycoplasma infection was significantly higher in previously reported cohorts of veterinarians, veterinary technicians, spouses of veterinary professionals, and others with extensive arthropod exposure and/or frequent animal contact. In the same study, *Mycoplasma ovis*-like species was the most prevalent organism detected. Therefore, the role of *M. ovis* should be further investigated, as it could pose a greater risk for public health, particularly for farmers and veterinarians, who are at higher risk of occupational exposure.

## 5. Conclusions

This study constitutes the first molecular evidence of hemotropic mycoplasmas in Ugandan cattle and goats and the entire East African region. Our results indicate a higher infection rate in goats than cattle. This study also suggests that *Candidatus* Mycoplasma erythrocervae can infect goats. Our study provides new information on the biodiversity of vector-borne pathogens in cattle and goat populations in Uganda. However, given the limited research on hemotropic mycoplasmas in Africa, further studies are required to clarify the pathogenicity and epidemiology of bovine and caprine hemoplasmas species and their impact on the livestock industry in Uganda.

## Figures and Tables

**Table 1:** Organisms, target genes, PCR oligonucleotide primers, amplicon size, and references for the PCR assays.

Organism	Target Gene	Primer Sequence (5′→3′)	Annealing Temperature (°C)	Amplicon Size (bp)	Reference
*Mycoplasma* spp. (HBT)	16S rRNA	ATACGGCCCATATTCCTACG	60	595	[26]
		TGCTCCACCACTTGTTCA			
*Mycoplasma* spp. (F2R2)	16S rRNA	CGAAAGTCTGATGGAGCAATA	60	170–-195	[25]
		CGCCCAATAAATCCGR(A/G)ATAAT			

**Table 2:** Overall detection rates of hemotropic mycoplasma species in cattle and goats.

Host	N	No. of Positives (%)	*p*-Value
Cattle	208	67 (32.2%)	0.019
Goats	201	88 (43.8%)	
Total	409	155 (37.9%)	

significant (<0.05).

**Table 3:** Hemoplasma detection rates based on sex and location.

		N	No. of Positives (%)	*p*-Value
Sex	Female cattle	193	58 (30.1%)	0.017
	Male cattle	15	6 (40.0%)	
	Total	208	64 (30.8%)	
Cattle	Karusandara subcounty	113	39 (34.5%)	0.459
	Kichwamba subcounty	95	28 (29.5%)	
	Total	208	67 (32.2%)	
Goats	Karusandara subcounty	103	56 (54.3%)	0.002
	Kichwamba subcounty	98	32 (32.6%)	
	Total	201	88 (43.8%)	

Significant (<0.05).

**Table 4:** GenBank accession numbers for the hemoplasma gene sequences obtained in this study.

Pathogen	Host	GenBank Accession Numbers
*Candidatus* Mycoplasma haemobos	Cattle	CMh1-7
*Mycoplasma wenyonii*	Cattle	Mw1-6
*Candidatus* Mycoplasma erythrocervae	Goat	CMe1
*Mycoplasma ovis*	Goat	Mo1-8

## Supplementary Materials

The following are available online at https://www.mdpi.com/2076-2615/10/9/1624/s1, Table S1: Additional information concerning sex, age, breed, body condition score, and herd size was obtained.
